# Sleep bruxism, awake bruxism and headache in children and adolescents: a scoping review

**DOI:** 10.22514/jofph.2024.034

**Published:** 2024-12-12

**Authors:** Marcela Carla Pereira do Nascimento, Thays Flavia Assis de Oliveira Melo, Rui Gonçalves da Luz Neto, Mariana Araújo Coutinho da Silveira, Sandra Conceição Maria Vieira, Mônica Vilela Heimer

**Affiliations:** ^1^Postgraduate Program in Dentistry, University of Pernambuco, 50100 010 Recife, PE, Brazil; ^2^Postgraduate Program in Hebiatrics, Faculty of Dentistry of Pernambuco, University of Pernambuco, 50100 010 Recife, PE, Brazil; ^3^Faculty of Dentistry of Pernambuco, University of Pernambuco, 50100 010 Recife, PE, Brazil

**Keywords:** Bruxism, Headache, Adolescent, Child

## Abstract

A scoping review was carried out with the aim of mapping the existing literature on the association between sleep/awake bruxism and primary headache (migraine and tension headache) in children and adolescents. This scoping review followed the method proposed by Arksey & O’Malley and the Joanna Briggs Institute Manual for Evidence Synthesis and was reported following the Preferred Reporting Items for Systematic Reviews and Meta-Analyses—Extension for Scoping Reviews (PRISMA-ScR). The methods were registered in the Open Science Framework (<osf.io/8mtv4 >). The following was the guiding question: “What does the literature say about the association between bruxism (sleep and awake) and primary headache (migraine and tension headache) in children and adolescents?”. Two independent researchers performed searches of the Cochrane Library, Embase, PubMed/Medline, Scopus and Web of Science electronic databases. Searches were conducted in August and September 2022 and updated in July 2023, leading to the retrieval of 6089 articles, 11 of which were selected for inclusion in the review. Sleep bruxism was associated with migraine as well as the frequency, duration, and intensity of migraine. Patients with tension headache are at increased risk for sleep bruxism and girls are more affected by both migraine and tension headache. In this scoping review, an association was found between primary headache (tension headache and migraine) and sleep bruxism. Awake bruxism was not investigated separately, making it difficult to determine its association with headache. The interaction between these variables is a complex phenomenon of unknown nature that merits further research.

## 1. Introduction

Bruxism is defined as a repetitive activity of the masticatory muscles characterized by clenching or grinding the teeth and/or bracing or thrusting the jaw and has two circadian manifestations. During sleep and in waking states, the condition is denominated sleep bruxism (SB) and awake bruxism (AB), respectively [1]. The international consensus on bruxism proposes three different diagnostic classifications. Bruxism is considered “possible” when based on a self-report or parental report, “probable” when based on a clinical assessment and “definitive” when based on an instrumental assessment, such as polysomnography (PSG) or electromyography [1].

Both SB and AB in children and adolescents have a multifactorial etiology with physical, psychological and environmental components. Common risk factors include stress, anxiety, sleep disorders, emotional issues and irregular sleep patterns, all of which should not be overlooked [2, 3, 4, 5]. The fact that bruxism can be influenced by a combination of these factors underscores the importance of a multidisciplinary treatment approach [6, 7, 8, 9]. It was reported a prevalence of 30% for SB [10], while for AB, a wide variation in prevalence rates was identified, from 4.1% to 37.3% [11].

The condition is related to psychological, exogenous and biological factors, especially in childhood [12, 13, 14]. Bruxism and temporomandibular disorder (TMD) have both been observed in individuals with episodic headache [15]. Indeed, some authors suggest an association between bruxism and headache [10, 16, 17, 18]. Headache, neck pain, insomnia, jaw pain, and difficulty opening the mouth are symptoms often detected in individuals with bruxism [19, 20, 21, 22].

Manifestations of headache differ in children compared to adults [23, 24]. Headache is the most common neurological complaint in childhood and adolescence, with a frequency of 58.4% in this population, and a higher incidence in females [25, 26]. A recent study conducted with adolescents showed that more than one-third of the sample characterized headache as disabling or of strong intensity, which raises concerns for parents and caregivers, as it can exert negative impact on the quality of life of children and adolescents [27, 28, 29, 30]. Headache is classified as primary or secondary. Primary headache is that in which pain symptoms define the condition, whereas secondary headache has an underlying cause [31, 32]. Tension-type headache (TTH) and migraine are frequent in children and adolescents [33]. One study found a positive association between bruxism (AB and SB) and the occurrence of headache three or more times per week, in addition to pain upon palpation in the masseter and temporal muscles in children and adolescents [34]. Other forms of primary headache are rarely observed in childhood [23].

Although the earliest studies on associations between bruxism and both TTH and migraine date back to 1959 and 1960, few epidemiological studies have focused on this population [35, 36]. Studies generally do not separate children and adolescents in the data analysis and most do not rely on standardized diagnostic criteria for assessing bruxism, which makes the level of evidence questionable [34], leading to high variability in prevalence rates. In the existing literature on the subject, most research has been conducted with adults, making it difficult to understand the manifestation of these conditions in children and adolescents [18, 34, 37, 38].

Therefore, the aim of the present study was to conduct a scoping review and include different study designs to map the existing literature on the association between bruxism (AB and SB) and headache. The secondary purpose was to identify existing gaps on the subject.

## 2. Method

This scoping review followed the five-step method proposed by Arksey and O’Malley [39] and the Johanna Briggs Institute Manual for Evidence Synthesis (2020). The writing of the article was guided by the Preferred Reporting Items for Systematic Reviews and Meta-Analyses—Extension for Scoping Reviews (PRISMA-ScR). The methods were registered in the Open Science Framework platform (<osf.io/8mtv4>).

### 2.1 Step 1: identifying the research question

The following research question was proposed: “What does the literature say about the association between bruxism (sleep and awake) and primary headache (migraine and tension-type headache) in children and adolescents?”.

### 2.2 Step 2: identifying relevant studies

Two reviewers (MCPN and TFAOM) independently performed searches of the Cochrane Library, Embase, PubMed/Medline, Scopus and Web of Science electronic databases for relevant studies. The Medical Subject Headings (MeSH) database was consulted to define the search terms: Bruxism/Sleep bruxism/Migraine/Headache/Tension-type headache/Child. The search strategy was adapted considering the specificities of each database (Table 1).

**Table 1.** Database search strategies.

Data base	Search strategy
Cochrane	Bruxism AND Headache AND Adolescent AND Child
Embase	Sleep Bruxism AND Adolescent AND Child AND Bruxism AND Headache OR Tension Headache
PubMed	Bruxism OR Sleep bruxism AND migraine AND headache OR tension-type headache AND Adolescent AND Child
Scopus	Bruxism AND migraine OR headache OR tension-type headache AND adolescent AND Child
Web of Science	Bruxism AND Migraine OR Headache AND Adolescent AND Child

A manual search was also performed in specific scientific journals that address the topic: Sleep Medicine, Headache: The Journal of Head and Face Pain, Brazilian Oral Research, Cephalalgia: An International Journal of Headache, European Journal of Oral Sciences and BMC Research Notes. No restriction was imposed with regards to the date of publication.

### 2.3 Step 3: study selection

The review inclusion criteria were based on the type of study. Articles from different study designs (observational studies and clinical research) were included. Study in children and adolescents up to 19 years old, the age range was based on the World Health Organization (WHO) definition of adolescence (10 to 19 years old) [40]. Articles that used different criteria for the diagnosis of bruxism (detection method questionnaire, clinical evolution or PSG) were included. Additionally, articles that report data on the relationship between bruxism and headaches, even if as a secondary objective. Therefore, articles that potentially met the review inclusion criteria were retrieved in full. No restrictions were imposed with regards to language or year of publication. The PCC strategy was used for the inclusion of studies in this review: P (population): children and adolescents; C (concept): bruxism; C (context): headache. Theses, dissertations, abstracts from scientific events, literature reviews, case reports and studies that involved adults were excluded.

After searching the databases, duplicate articles were excluded using the Mendeley Reference Manager tool (Mendeley Ltd., Elsevier). Applying the eligibility criteria, two independent reviewers (MCPN and TFAOM) preselected articles based on an analysis of the titles and abstracts. Agreement between the reviewers was determined using the Kappa index, which indicated nearly perfect agreement (K = 0.96) in all databases. To determine inclusion, each preselected article was read in its entirety by both reviewers.

One reviewer (MCPN) extracted the data and another (TFAOM) reviewed the results. In all stages, disagreements were resolved through discussion with a third reviewer (MVH). A search was also performed of the reference lists of the articles included in this review to identify possible studies not retrieved during the search of the databases.

### 2.4 Step 4: data extraction

The following data were extracted from the articles selected: authors/year, location, study design, sample/age group, instruments, objectives, results and conclusion. Following data extraction, a synthesis was performed using thematic analysis related to the guiding question of this review. Coding and initial analysis were performed by two reviewers who discussed the content of the themes and subthemes, reaching a consensus after the second meeting.

### 2.5 Step 5: collating, summarizing, and reporting the results

A flowchart was created to illustrate the article selection process, as proposed by the PRISMA-ScR (Fig. 1). The data are presented in a tabular form with the individual results of each study (Table 2).

**Fig. 1.** PRISMA flowchart.

**Table 2.** Characteristics of studies included in present review.

Author/Year/Country	Study design	Sample Age group (yr)	Instruments	Aim	Results	Conclusion
Yaghini *et al*. [41] (2010) Iran	Cross-sectional	148 6–14 yr	Examination by a neurologist, sociodemographic questionnaire and 10 sleep questions.	To assess the relative frequency of sleep disturbances in children with and without migraine.	Association between migraine and SB (*p* = 0.04).	Sleep bruxism was detected significantly more in children and adolescents with migraine compared to children and adolescents without migraine.
Bruni *et al*. [42] (1997) Italy	Control case	238 (164 migraine and 119 tension-type headache)	Questionnaire completed by parents addressing clinical history, sleep disorders, sleep habits, and sociodemographic data.	To determine the prevalence of sleep disorders, migraine and tension-type headache in children and adolescents.	SB was identified in 7.4% of controls, 12.2% of individuals with migraine and 7.6% of individuals with tension-type headache, demonstrating a significant difference in the migraine group (*p* < 0.05).	Association between SB and migraine.
Masuko *et al*. [43] (2014) Brazil	Case-control	20 controls and 20 cases 6–12 yr	Polysomnography using the Alice 3 System. SB criteria were in accordance with American Academy of Sleep Medicine (AAMS), 2007.	Use polysomnography to investigate the prevalence of SB in children and adolescents with episodic migraine versus controls.	25% of children with sporadic migraine had bruxism during the sleep study (*p* = 0.045).	SB is more prevalent in patients with episodic migraine. None had migraine with aura.
Miller *et al*. [44] (2003) USA	Cross-sectional	118 2–12 yr	Sleep habits questionnaires, headache assessment questionnaire according to International Headache Society (IHS) criteria.	Investigate the prevalence of sleep disorders in children and adolescents with migraine and describe differences in sleep behaviors based on headache characteristics.	Migraine frequency positively correlated with SB (*r* = 26, *p* < 0.001).	Frequency, duration, and intensity of migraine-associated pain related to the occurrence of specific sleep-related behaviors, including SB.
Liljeström *et al*. [45] (2001) Finland	Case control	297 13–14 yr 66 controls and 231 cases	Questionnaire, examination by physician and physiotherapist, and dental examination.	Investigate the association between different types of headache (migraine, episodic tension-type headache, chronic tension) and parafunctions, such as sleep bruxism (SB).	No statistically significant association between headache and SB (*p* > 0.05).	Despite absence of association between SB and tension-type headache, SB was reported more by children with tension-type headache.
Vendrame *et al*. [46] (2008) USA	Cross-sectional	90 with headache 5–19 yr	Polysomnographic records, examination, and review of medical records.	To characterize polysomnographic findings in children with headache (migraine, chronic migraine, tension-type headache, non-specific headache) and sleep problems; describe differences in sleep architecture of children in relation to headache diagnoses.	50% of participants with tension-type headache expressed sleep bruxism, while only 2.4% of patients without headache manifested sleep bruxism (odds ratio = 1.95; 95% confidence interval: 1.2–4.34).	Patients with tension-type headache had two-fold increased risk of developing SB compared to those with non-tension-type headache group.
Carra *et al*. [47] (2011) Canada	Cross-sectional	604 7–17 yr	Questionnaire divided into four sections (medical, dental, sleep and awake bruxism (AB), temporomandibular disorder (TMD), sleep disorders), clinical examination, and orthodontic assessment.	Investigate association between SB/AB and signs and symptoms of TMD, sleep problems, and behavioral complaints in a population seeking orthodontic treatment.	12.1% of subjects with SB had more frequent headache compared to 4.1% of controls (*p* = 0.05).	Children and adolescents with SB had headache three times more often than controls.
Wanman and Agerberg *et al*. [48] (1986a) Sweden	Cohort	285 aged 17 yr	Dental examination, questionnaire general health status, allergies, sleep disorders, parafunctional habits, such as bruxism; for headache, a 5-degree scale for both frequency and intensity.	Assess the frequency and intensity of headache and symptoms of masticatory system dysfunction.	Frequency of headache with teeth grinding (AB) (*p* > 0.01) Frequency of headache with teeth grinding (SB) (*p* < 0.001). Frequency of headache with clenching and/or grinding (*p* < 0.001).	Habits such as grinding and clenching teeth were related to headache frequency, but not intensity.
Wänman and Agerberg *et al*. [49] (1986b) Sweden	Cohort	285 aged 17 yr	Questionnaire and clinical examination.	Prevalence of TMD signs and symptoms in adolescents.	Prevalence of headache (*p* < 0.001) and teeth grinding (*p* < 0.05) higher in girls.	Patients with high prevalence of sleep bruxism also had high prevalence of headache, with females affected more by both conditions.
Nilner *et al*. [50] (1983) Sweden	Cross-sectional	309 15–18 yr	Interview and Clinical Examination.	To investigate the association between oral parafunctions and functional disorders of the stomatognathic system.	A strong correlation was found between bruxism and headache (*p* < 0.01).	Bruxism appears to play an important role in the etiology of muscle tenderness and recurrent headache.
Atsü *et al*. [51] (2019) Turkey	Cross-sectional	270 15–18 yr	Questionnaire, clinical examination and two psychometric tests.	To investigate associations between oral parafunctions, personality traits, anxiety and signs and symptoms of temporomandibular disorders in adolescents.	Data analysis revealed that bruxism was associated with headache (*p* < 0.05).	Adolescents who suffered from bruxism were 2.89 times more likely to have tension-type headache.

## 3. Results

The bibliographic survey was performed in August and September 2022 and updated in July 2023 based on published and indexed records in the databases used. The database searches and manual searches led to the retrieval of 6089 articles, 1931 of which were duplicates and were removed. Among the remaining 4158 articles, the analysis of the titles and abstracts led to the exclusion of 4114, leaving 44 for full-text analysis, 33 of which were excluded for different reasons, leaving 11 articles to compose this review (Fig. 1).

With regards to geographical distribution, 45.4% of the studies were conducted in Europe, 36.3% in the Americas, 9% in Asia, and 9% in Turkey (transcontinental country). Six studies had a cross-sectional design, two were cohort studies, and three were case-control studies. No randomized clinical trials on the subject were found.

Table 2 summarizes the studies on bruxism (SB and AB) and primary headache (migraine and tension-type headache) included in this scoping review, with descriptions of the following data: authors, year, country, study design, study population, instruments, aims, results and conclusion.

## 4. Discussion

The results of this review showed an association between bruxism and headache. However, only five studies specified the type of primary headache and the circadian manifestation of bruxism [41, 42, 43, 44, 45]. Some studies found an association between SB and migraine [41, 42, 43, 44]. A study conducted in 2008 found an association between SB and tension-type headache [46]. Other studies reported as association between SB and headache, but with insufficient information on the characteristics of headache [47, 48, 49, 50, 51]. A study conducted in the 2000s addressing SB and primary headache was the only study not to find an association between the variables [45].

Self-administered questionnaires were the most widely used instruments [41, 42, 44, 47, 50]. Only two studies involved PSG for the diagnosis of SB [43, 46]. Both studies had smaller samples, as the polysomnographic exam, which is the gold standard for investigating bruxism, is expensive and has limited access in some places [18, 52]. Thus, in studies involving children and adolescents, self-reports or the reports of parents and guardians combined with an intraoral clinical examination are widely used for the assessment of bruxism [1, 45]. It is noteworthy that none of the studies included in this review provided information on the frequency and intensity of bruxism episodes.

Among the eleven articles that composed this review, three reported causality in the relationship between bruxism and headache [42, 43, 49]. Two of these studies pointed to the existence of a common pathogenic pathway between the phenomena. The hypothesis was the presence of chronic sympathetic dysregulation [42, 43]. Another important finding was that teeth grinding and clenching induced facial pain and increased blood flow to the muscles, leading to fatigue, sensitivity and muscle pain, which may be one of the mechanisms of headache [49].

With regard to headache, especially primary headache, neurological assessments, self-administered questionnaires with a scale of headache frequency and intensity and the patient’s previous history were listed as criteria [41, 42, 43, 44, 46, 48]. Even when using specific diagnostic criteria, diagnosing headache is difficult due to the possibility of diagnostic overlap, such as TTH resulting from muscle tension attributed to TMD [44]. In the study by Nilner [50], the participants experienced headache and pain in the temples correlated with palpation sensitivity related to the temporomandibular joint and associated muscles.

The interaction between TTH and SB was investigated in a study conducted in 2008 [46]. TTH was found to be a risk factor for bruxism, as patients with TTH were twice as likely to develop the parafunctional habit of bruxism compared to patients with non-tension headache. It should be noted that this research involved an intentional sample, which should be considered a limitation when interpreting the findings.

In one study, children and adolescents with migraine were 1.6 times more likely to have bruxism compared to those without this complaint [44]. When examining the characteristics of migraine, the authors found that the interaction occurred not only between migraine and SB, but also other sleep disorders, leading to the assumption that sleep disorders and migraine may have common genetic and pathophysiological components [41, 42, 43, 44].

From another perspective, Atsü *et al*. [51] carried out a cross-sectional study with a non-probabilistic sample and found that individuals who suffered from SB were 2.89 times more likely to develop headache compared to those who do not have this sleep disorder. This association may be explained by the characteristics of SB, as the condition is conceived as an increase in the frequency of masticatory muscle activity during sleep, which leads to micro-arousals and fragmented sleep, with a reduction in total sleep time [10]. Indeed, insufficient and non-restorative sleep have been reported to be risk factors for headache [53].

According to two longitudinal studies that shared the same sample but had different objectives, girls suffer from headache more than boys [48, 49]. This finding is consistent with results described by Luvsannorov *et al*. [54] (2021) and Okamura *et al*. [55] (2020), who found that individuals with headache were predominantly female. This is believed to be a consequence of hormonal variations, such as premenstrual tension. In these longitudinal studies, an association was found between SB and the frequency of headache but no association was found with the headache intensity, which may be explained by the age group analyzed, as only 17-year-old adolescents were included in the sample [48, 49]. In contrast, a significant association was found between SB and headache frequency [44, 48, 49, 56]. These findings are in line with results described in the study by Sousa *et al*. [57], who concluded that individuals diagnosed with SB suffer from headache more often, especially upon waking.

The number of studies on SB is much higher compared those addressing AB, which is often not addressed separately, but together with SB. Furthermore, it is still unclear whether SB and AB share the same clinical characteristics Orofacial pain in children and adolescents is believed to be more associated with AB. Indeed, recent studies report that children and adolescents with orofacial pain are more likely to have AB, whereas tooth wear and headache are more prevalent in cases of SB [34]. Considering the negative impact of AB on quality of life, which may be associated with bullying [58, 59], studying this condition is a way to clarify the phenomena involved in its emergence, thus contributing to the improvement of the well-being of the child and adolescent population. It is noteworthy that bruxism can be an important contributing factor to the development of trigger points in the head and neck, which, in turn, generate and/or contribute to (TTH), as tooth contact is frequent and intense [60]. However, when investigating the interaction between bruxism and primary headache (migraine and TTH), an association was only found with migraine [42].

As demonstrated in the present review, few studies address the topic of “bruxism and headache” in childhood and adolescence, constituting a gap in the literature. Moreover, intentional and nonrepresentative samples were found, along with a wide variety of diagnostic criteria, making comparisons between studies difficult. The predominance of the cross-sectional design is also noteworthy, demonstrating the need for longitudinal studies that can provide a better understanding of the association between the variables.

## 5. Conclusion

Based on the findings of this scoping review, the following conclusions were drawn:

• A significant association was found between the frequency of headache and sleep bruxism, but the association with headache intensity remains unclear;

• Awake bruxism was not addressed individually, which prevents determining its association with headache;

• The data lead to a reflection that there may be a common physiopathological basis between bruxism and headache;

• The nature of the interaction remains unknown, requiring further investigation, especially with regards to awake bruxism due to the small number of studies on this variable;

• Few studies have addressed the topic “bruxism and headache” in childhood and adolescence, characterizing a gap in the literature.

## 6. Highlights

• Intentional and nonrepresentative samples were found.

• A wide variety of diagnostic criteria have been employed, making comparisons between studies difficult, which hinders the generation of robust evidence for healthcare providers.

• The predominance of cross-sectional studies underscores the need for longitudinal designs that can provide a better understanding of the association between bruxism and headache.
